# Automating Large-scale Health Care Service Feedback Analysis: Sentiment Analysis and Topic Modeling Study

**DOI:** 10.2196/29385

**Published:** 2022-04-11

**Authors:** George Alexander, Mohammed Bahja, Gibran Farook Butt

**Affiliations:** 1 The School of Computer Science University of Birmingham Birmingham United Kingdom

**Keywords:** natural language processing, topic modeling, National Health Service, latent Dirichlet allocation, reviews, patient feedback, automated solutions, large-scale health service, free-text, unstructured data

## Abstract

**Background:**

Obtaining patient feedback is an essential mechanism for health care service providers to assess their quality and effectiveness. Unlike assessments of clinical outcomes, feedback from patients offers insights into their lived experiences. The Department of Health and Social Care in England via National Health Service Digital operates a patient feedback web service through which patients can leave feedback of their experiences in structured and free-text report forms. Free-text feedback, compared with structured questionnaires, may be less biased by the feedback collector and, thus, more representative; however, it is harder to analyze in large quantities and challenging to derive meaningful, quantitative outcomes.

**Objective:**

The aim of this study is to build a novel data analysis and interactive visualization pipeline accessible through an interactive web application to facilitate the interrogation of and provide unique insights into National Health Service patient feedback.

**Methods:**

This study details the development of a text analysis tool that uses contemporary natural language processing and machine learning models to analyze free-text clinical service reviews to develop a robust classification model and interactive visualization web application. The methodology is based on the design science research paradigm and was conducted in three iterations: a sentiment analysis of the patient feedback corpus in the first iteration, topic modeling (unigram and bigram)–based analysis for topic identification in the second iteration, and nested topic modeling in the third iteration that combines sentiment analysis and topic modeling methods. An interactive data visualization web application for use by the general public was then created, presenting the data on a geographic representation of the country, making it easily accessible.

**Results:**

Of the 11,103 possible clinical services that could be reviewed across England, 2030 (18.28%) different services received a combined total of 51,845 reviews between October 1, 2017, and September 30, 2019. Dominant topics were identified for the entire corpus followed by negative- and positive-sentiment topics in turn. Reviews containing high- and low-sentiment topics occurred more frequently than reviews containing less polarized topics. Time-series analysis identified trends in topic and sentiment occurrence frequency across the study period.

**Conclusions:**

Using contemporary natural language processing techniques, unstructured text data were effectively characterized for further analysis and visualization. An efficient pipeline was successfully combined with a web application, making automated analysis and dissemination of large volumes of information accessible. This study represents a significant step in efforts to generate and visualize useful, actionable, and unique information from free-text patient reviews.

## Introduction

### Background

Patient experience is described by the Beryl Institute as “the sum of all interactions, shaped by an organisation’s culture, that influence patient perceptions across the continuum of care” [1]. It is a vital consideration of the health service provider’s planning strategy to reflect patient engagement and service quality [2]. It is also a contributing factor to patient engagement, which is key to delivering effective and efficient care [3-6]. The *Patient experience improvement framework* of the National Health Service (NHS) defines several quality indicators, among which patient feedback is a priority [7]. Thus, to deliver truly patient-centered care, the patient experience must be a central consideration [8], and health care providers must have mechanisms in place through which the patient experience can be understood.

Over the past 2 decades, there has been a greater emphasis on using patient feedback to inform and improve service delivery, largely in the United Kingdom, Europe, and the United States [9,10]. The way feedback is obtained can vary greatly, ranging from individual interviews and focus groups to official complaints as well as surveys conducted through various media (postal or web-based). Surveys and similar quantitative methodologies generate measurable results that can be used for benchmarking and comparisons over time or between subjects. Although they can help identify some problem areas in services, they can lack the specificity required to drive change [10-12]. Feedback mechanisms that allow in-depth ideas to be shared, such as patient forums, can help generate detailed patient experience insights [10-12].

The NHS website allows users to anonymously rate and share their experience in a public forum [13]. All NHS-provided services across England can be found on the site, where users can leave a free-text comment and give an overall *star* rating out of 6. These publicly available reviews are invaluable insights into the work of the NHS for the service providers themselves, national bodies such as NHS England, and the Care Quality Commission as well as patients deliberating on which services to use. This is a source of vast amounts of rich data, which has the potential to significantly influence the quality of services nationwide as well as policy regarding the NHS. Patient feedback in free-text form is typically hard to analyze on a large scale, which is why standardized scales are more frequently used to generate numerical measures for comparisons [14]. The difficulty from the patients’ perspective lies in the accessibility to the data, which is limited to scrolling through individual responses in a particular service.

This type of data lends itself well to analysis using computed natural language processing (NLP) techniques, enabling high-volume automated analysis of text information. In their seminal work, Greaves et al [15] reported on the utility of this NHS web-based feedback data to gain insights into the health care service while allaying concerns about the risk of unsolicited reviews creating biased feedback. Greaves also reported that, with regard to the accuracy of the feedback about a given clinical service, web-based feedback was comparable with conventional surveys of patient experience [15].

The advent of machine learning and the development of sophisticated NLP algorithms have significantly advanced the analysis of text corpora. A significant amount of research has focused on applying advanced NLP methods to web-based reviews. Web-based reviews provide an opportunity to explore free-text corpora that do not usually adhere to a structure or format. The free text in reviews, such as the patient experience, makes the process of automated analysis of the review challenging when compared with closed questions with an expected text input. As web-based patient feedback is extensive, the traditional text analysis methods may provide limited capabilities for analysis. The latest machine learning– and artificial intelligence–based NLP methods have been well explored for analyzing large review data sets, especially for analyzing the user experience. The latest NLP methods provide capabilities to classify the reviews as positive or negative with high accuracy. Identifying the underlying themes and topics in the user experience allows us to understand the frequently reported service areas in user feedback.

### Objective

The aim of the study presented in this paper is to provide an automated solution for the large-scale analysis of patient feedback on health care service providers. This study achieves this by exploring NLP techniques, including sentiment analysis and topic modeling. The objective of this study is also to present an interactive interface that provides stakeholders with a portal to analyze and identify outcomes of the patient feedback analysis. This work builds on previous work in the field [16,17] and presents the design and implementation of an unsupervised machine learning NLP model combined with an interactive interface to produce a user-friendly web application that allows for the exploration of service reviews across England.

## Methods

### Overview

For this study, the design science research (DSR) methodology was used. The DSR paradigm is a widely popular research approach in information systems research. It is referred to as a problem-solving paradigm as it aims to build *artifacts* that are aimed at addressing a problem. The artifacts address the problems or enhance existing solutions and are important tools for arriving at research outcomes and reviewing them to decide how the adopted artifact can be further used [18]. The DSR process follows a systematic procedure in which the artifacts are developed through the systematic creation, capturing, and communication of knowledge from the design process. DSR uses an iterative process whereby the artifacts are reconstructed at each iteration and, thus, can be described as a continuous learning process that enhances the artifact quality incrementally [19]. Further details on the DSR methodology in this study can be found in our previous study [17].

**Figure 1:**
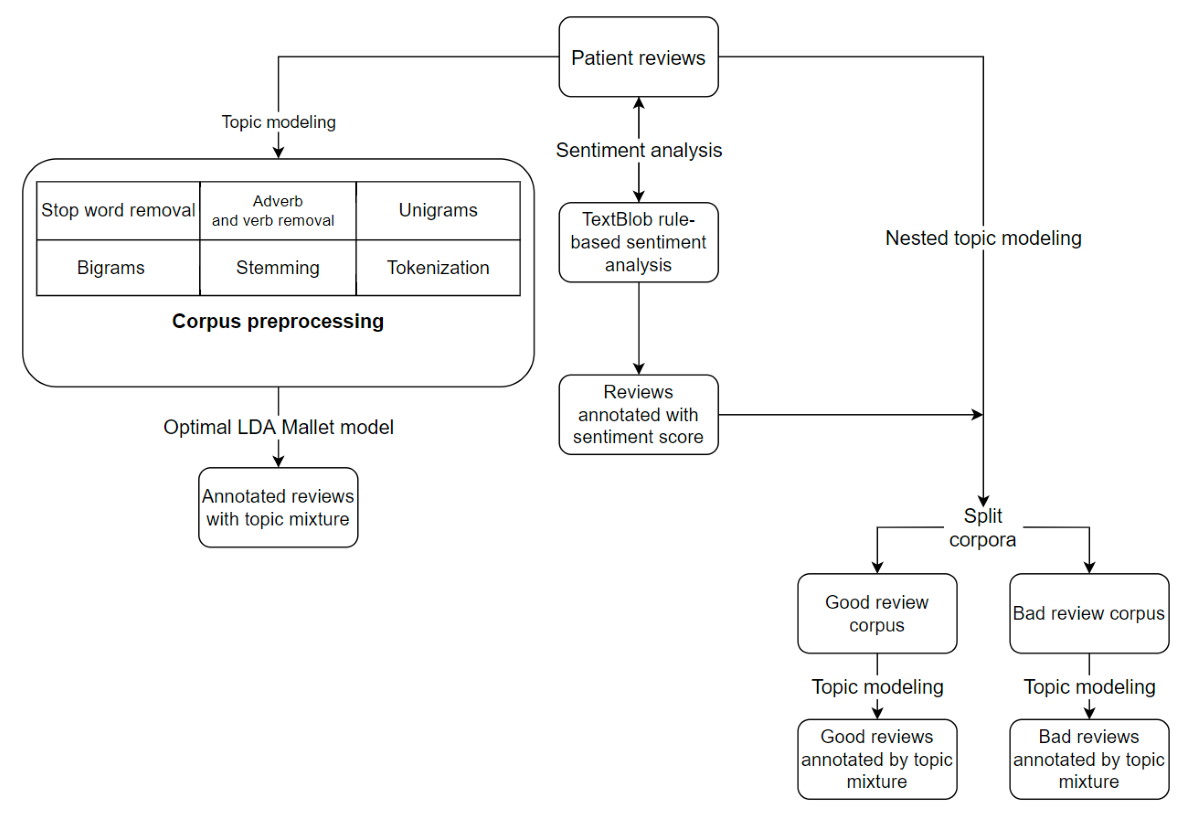
The research methodology followed for the analysis of the patient feedback corpus. LDA: latent Dirichlet allocation.

Figure 1 illustrates the methodology carried out for this research. The patient review corpus was subjected to three different iterations of NLP analysis: sentiment analysis, topic modeling, and nested topic modeling. The first iteration, sentiment analysis, enabled the automated analysis of the patient reviews and identified the sentiment of the reviews as either positive or negative. The second iteration, topic modeling analysis, applied the unigram and bigram topic modeling methods to annotate reviews with a group of words that reflected a theme or topic. A single review might have one or more topics. In the third iteration of the study, a nested topic modeling approach was applied. Nested topic modeling analysis combines sentiment analysis and topic modeling methods. The patient reviews were annotated with their associated sentiment score and then split into the *good* corpus and *bad* corpus based on the associated positive or negative sentiment. The *good* corpus and *bad* corpus were analyzed with topic modeling to identify topics within the reviews.

### NHS Patient Feedback

The NHS website includes a platform where patients provide both ratings and reviews for a particular NHS service. The NHS website rating system provides an outline of patient experience; the rating is an optional feature that is collected for a specific set of parameters such as *cleanliness* and *dealt with dignity*, among others.

Patients can provide feedback about NHS hospitals in 3 main sections. First, they are asked to rate, on a scale of 1 to 6, *how likely they are to recommend the particular hospital to family and friends?* This is the central question on the NHS website in that the ratings provided are used to calculate the overall rating for a given hospital. The rating for this question provides a quick and easy indicator of a hospital’s performance in providing patient care. However, the rating is single and straightforward, and it is insufficient to obtain a detailed understanding of hospital performance. For a more detailed understanding of patient feedback, the NHS website includes questions where the patients are asked to provide ratings on five parameters: cleanliness, staff co-operation, dignity and respect, involvement in decisions, and same-sex accommodation (out of 6 stars). The website-allocated ratings for these 5 parameters are optional to the users. Finally, there is an optional free-text review of a maximum of 3000 characters. These reviews follow the NHS comment policy and are moderated. The moderators remove any personal information and ensure that the reviews do not cause any legal issues such as defamation [13]. Responses to the 5 parameters and free-text data provide an opportunity for a granular assessment and understanding of patient feedback for a hospital.

All available reviews between October 1, 2017, and September 30, 2019, were collected from the NHS website.

The NHS platform provides an application programming interface (API) that allows access to the patient ratings and reviews [20]. A custom web scraper was built for the project using the NHS API to collect the patient ratings and reviews. The NHS platform provides the data with the standard license terms that cover the requirements of the General Data Protection Regulations [21].

### Data Preprocessing

The data set underwent a few processing steps where only columns from the data set that were relevant for this study were selected. Specifically, for parsimony purposes, only relevant data fields were extracted and relabeled into *Date*, *Comment*, and *Label* columns in the database. The *Date* and *Comment* columns referred to the posted date and the content of the participant’s comment, and the *Label* column was used to hold the sentiment of the comment, classified as either positive or negative.

Data were organized by posting date [22,23] and partitioned into training, test, and validation data sets. In the training data set, observations were labeled according to the sentiment inferred from the comments. The test data set was used as an input to derive patterns from the training data set using text-mining models. In the validation data set, the values in the *Label* column were not defined.

Within the data set, the ratings given by the participants for the following question—*How likely are you to recommend this hospital to friends and family if they needed similar care or treatment?*—were used as the actual data against which the performance of the text-mining model (ie, in predicting the patient feedback) was tested. Owing to the skewed distribution of the numerical responses and limitations of the machine learning methods, to reduce complexity in the modeling procedure, the continuous-scale patient feedback ratings were discretized. Following discretization, rating scores of 1 and 2 were categorized as negative, and those of 5 and 6 were categorized as positive. Rating scores of 3 and 4 were discarded as they were deemed neutral ratings that did not portray polarization. This categorization served as a binary sentiment label for which each text-mining model was trained and assessed.

### Sentiment Analysis

Sentiment analysis, also referred to as opinion mining, refers to the computational study of people’s opinions, sentiments, attitudes, and emotions toward an entity. The entity can be another individual or a public figure, a product such as an electronic device, or service providers such as restaurants and hospitals. The identification of sentiment is performed based on the presence of words or phrases that are likely to refer to an opinion, sentiment, or emotion. If the sentence is identified as having a sentiment or opinion, it is then subjected to the feature selection process. During this process, the identified sentiment is associated with the feature that is being discussed. Finally, the sentiment is categorized into a chosen classification type. For instance, the sentiment can be classified into a binary, such as positive or negative. The sentiment analysis is associated with a score.

The fine-grained sentiment score detects polarity within a text; in this case, whether the review is a positive or negative opinion. There are several approaches to sentiment analysis, including the strength of association, naïve Bayes (NB), and the support vector model. NB-based sentiment analysis models are popular and widely used. The NB classifier is a probabilistic classifier, which uses a mixture of models for classification and is widely popular for sentiment classification. Given a document, and based on the distribution of words in the document, the NB approach computes the probability of a document belonging to a class. This model calculates boundaries according to the distribution of the words across the labels while at the same time considering the joint probability of the words occurring independently together. Specifically, NB considers each word independently of one another and then tries to estimate the posterior distribution of a review being positive or negative according to the joint distribution of the words in the review. The probability is computed using the Bayes theorem to predict that a given word belongs to a specific sentiment.

One of the popular implementations of sentiment analysis based on NB is the TextBlob rule-based sentiment analysis [24], which was adopted in our study. The TextBlob approach allows for the performance of different NLP tasks, including part-of-speech tagging, noun phrase extraction, sentiment analysis, classification, and translation. The TextBlob approach is suitable in the current version of our study when compared with advanced machine learning–based approaches because of the relatively smaller size of the data set that is used. A machine learning–based method inherently requires large, labeled data sets for training and testing that could be prohibitive and expensive [25].

The TextBlob implementation was used to analyze each of the 51,845 reviews to determine their sentiment value. This produced a score for each review between −1 and 1, where 1 represents a wholly positive sentiment, −1 represents an entirely negative sentiment, and 0 represents a neutral sentiment. This provided a method for evaluating whether reviews were *good* or *bad* more quickly and consistently than a human-based process.

### Topic Modeling

Topic modeling is an unsupervised NLP approach where unlabeled documents are used to create a set of topics represented by a list of words that frequently occur in each topic. There are several topic modeling approaches, and most use dimensionality-reducing techniques with the goal of representing a document using fewer words. Some of the most popular topic modeling approaches are probabilistic latent semantic indexing (LSI), latent Dirichlet allocation (LDA), and correlated topic modeling (CTM).

The LSI approach uses linear algebraic approaches such as singular value decomposition and *bags of words* to represent documents. It aims to extract words that carry similar meanings (ie, it uses synonyms and polysemy for topic identification [26]). The LSI approach assumes that each document has multiple topics and that the probability of each consists of a weight for a given document. On the basis of this assumption, the topics in a document are identified.

A disadvantage of the LSI approach is that the number of parameters in the model increases as the volume of data increases, and this could lead to overfitting problems. Furthermore, when the LSI model is used on documents that were not part of the training data set, the topic probabilities have to be assigned again [27].

The CTM approach helps in identifying the correlation between a specific topic and others. The correlation information might help the users in identifying links or associations between a specific topic from a database and other similar topics. The CTM approach uses a logistic normal distribution to identify topics from documents. A covariance matrix used for parameterizing the distribution is then used to identify the correlation between the topics. A topic graph is subsequently drawn, in which the topics are represented by nodes and their correlations with other topics are depicted [28]. The correlation approach provides more information to the user and, thus, enables better interpretation of the information. The CTM approach achieves a higher predictive likelihood than the LSI approach [29].

The LDA approach also works under the assumption of LSI (ie, that each topic is a distribution of words and each document has a certain distribution of topics). However, this assumption is extended by using a hidden variable model of documents that consists of hidden random variables with which the observed data interact [30]. In LDA, the hidden variables are the topics and how the document exhibits them, and the observed data are the words. The learned or posterior distribution of the hidden variables for the given documents determines their topical composition. Furthermore, the LDA approach uses the Dirichlet distribution to define the distribution of topics in a document [31].

An advantage of the LDA approach is that the statistical assumptions it makes for topic modeling enable it to uncover sophisticated structures in the texts. For instance, the *bag of words* assumption used in the LDA approach makes it invariant to the order of words in the document. Furthermore, the order of documents in the corpus is also not a criterion for the LDA approach to extract topics from the document. This might not be suitable if the patient’s experience needs to be analyzed longitudinally (ie, over a period). However, in this study, as the patient experience analysis did not consider the time factor, the LDA method suited its aims.

We used the LDA topic modeling approach to categorize each review into computer-generated topics. LDA initially assumes the number of topics and attempts to calculate topics that best represent the documents. It does this by calculating the probability estimate of a word for a given topic as well as the probability of a topic for a given document [32].

We used an LDA implementation called LDA Mallet owing to its use of a more precise sampling method called Gibbs sampling [33]. Data were preprocessed, including the removal of stop words, verbs, and adverbs as well as lemmatization, formation of bigrams, and conversion of the corpus into the bag-of-words format. Bigrams were generated using the Gensim library (RARE Technologies, Ltd), which automatically detects common phrases [34]. LDA Mallet has 2 parameters, a number of topics, and a hyperparameter α. These parameters were optimized through a series of experiments, with the number of topics ranging from 5 to 25 and α ranging from .01 to .99. Each test was measured using the coherence score [35] as well as using human judgment to determine the validity of the generated topics. The highest-rated LDA model was then used to determine the topic mixture of each review.

Reviews were then labeled according to the dominant topic. The dominant topic was defined by the LDA model, predicting the percentage contribution of that topic to be ≥50%. The reviews that did not have any topic that contributed ≥50% were not included in the following analysis.

### Nested Topic Modeling

The corpus was divided into two subcorpora, the first one being negative-sentiment–scoring reviews and the second one being any positive-sentiment–scoring reviews. Similar to the topic model for the entire corpus, we then performed experiments to determine the optimal parameters for both corpora. Using these parameters, we produced two models, one showing the topics generated from negative-sentiment reviews and the other showing topics generated from positive-sentiment reviews. Applying these new LDA models to their respective corpora produced a topic mixture for each entry of their respective corpora.

The nested topic modeling approach enables the identification of the rationale behind a particular sentiment of the patient in each comment or review. The intent is to find out why the patient was happy or unhappy about a particular topic in each comment. The problem being addressed in this iteration of the study was to find the possible reason behind a patient’s sentiment for a particular topic in each comment.

### Visualization

Visualizing the results from the NLP methods relied on a Microsoft Azure Cloud Service [36] to host the SQL server, web functions (API), and the web application. The SQL server stored each review and service as well as the results from both the sentiment analysis and topic modeling. The web functions acted as an API to allow for a Representational State Transfer and secure connection to the database. Finally, the cloud service hosted the web application, which used NodeJS (OpenJS Foundation) [37] as the back-end framework and VueJS [38] as the front-end framework. The web application also used packages such as Google Maps [39] and VueChartJS [40]. Both of these packages ensured that the results were shown in a logical, effective, and efficient way.

## Results

### NHS Patient Feedback

NHS England is segmented into seven geographical regions with teams supporting the delivery of care locally: London, Midlands, North East and Yorkshire, North West, East of England, South East, and South West. Of the 11,103 possible services across these regions, 2030 (18.28%) services received a combined total of 51,845 reviews between October 1, 2017, and October 31, 2019. Among the reviewed services, the mean number of reviews per service was 26 (SD 60.3), and the highest number of reviews for a single service was 550 for Lincoln County Hospital. During the study period, the mean number of reviews per month across England was 2028 (SD 449.1). The number of reviews per month declined from 2625 in October 2017 to 1611 in September 2019.

The number of services per 10,000 population was similar around England, with the lowest being in London, which also has the highest-density population (Table 1). Across England, 18.11% (2011/11,103) of the services received a review during the study period, with the fewest services reviewed being in the North East and Yorkshire and the most reviewed being in London. The number of reviews per 10,000 population across England was similar, with the fewest being in the South East region and the highest in the East of England (Table 1).

**Table 1:** National Health Service England regions, services, and reviews.

Characteristic	England	East of England	London	Midlands	North East and Yorkshire	North West	South East	South West
Population (million)^a^	53.8	4.5	8.2	10.1	7.9	7.1	8.6	5.3
Services, n (%)	11,103 (100)	1095 (9.9)	1343 (12.1)	2123 (19.1)	1865 (16.8)	1572 (14.2)	1929 (17.4)	1176 (10.6)
Reviewed services, n (%)	2011 (18.1)	221 (20.2)	309 (23)	369 (17.4)	299 (16)	269 (17.1)	323 (16.7)	221 (18.8)
Unreviewed services, n (%)	9092 (81.9)	874 (79.8)	1034 (77)	1754 (82.6)	1566 (84)	1303 (82.9)	1606 (83.3)	955 (81.2)
Total reviews, n (%)	50,707 (100)	5517 (10.9)	8337 (16.4)	10,047 (19.8)	7212 (14.2)	6896 (13.6)	7709 (15.2)	4989 (9.8)
Reviews per 10,000	9.4	12.3	10.2	9.9	9.1	9.7	9.0	9.4
Services per 10,000	2.1	2.4	1.6	2.1	2.4	2.2	2.2	2.2

^a^Population data from the Office for National Statistics Census, 2011.

### Sentiment Analysis

**Figure 2:**
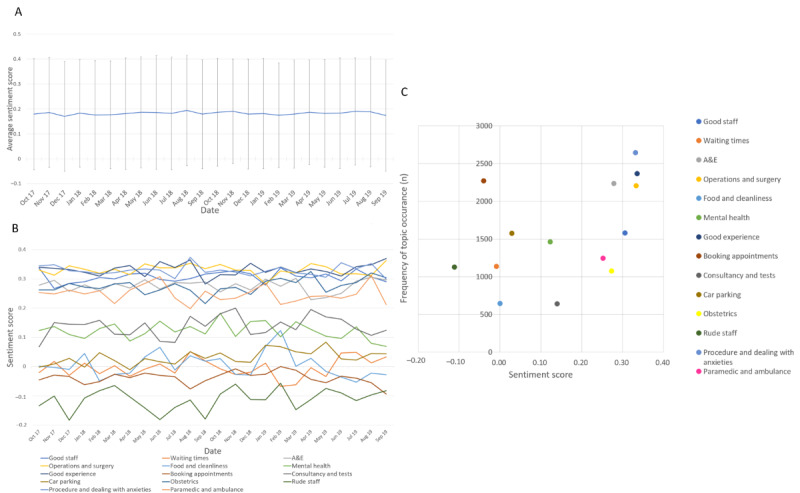
A collection of relationships within the data set. A: sentiment score over time; B: Relationship between topic frequency and sentiment score over time; C: Relationship between topic frequency and sentiment. A&E: accident and emergency.

To explore the opinions held within the reviews, the sentiment was analyzed for the entire corpus and for individual reviews. The analysis generated a score for how positive (eg, happy or pleased) or negative (eg, unhappy or disappointed) the sentiment was between −1 (most negative) and 1 (most positive). Examples of reviews and their corresponding score can be found in Table 2. The average sentiment of all reviews across the study period did not demonstrate any significant changes (Figure 2A). A comparison of sentiment by season revealed that there were no significant differences among spring (March 20 to June 21), summer (June 21 to September 22), and autumn (September 22 to December 21); however, a statistically significant decrease occurred in winter (December 21 to March 20; mean sentiment 0.178, SD 0.21) compared with summer (mean sentiment 0.185, SD 0.21; *P*=.02).

Table 3 reports the sentiment scores for each of the topics in the corpus. Topics that are innately associated with positive sentiments, such as good experience and good staff, have higher sentiment scores than inherently negative topics such as rude staff. The distribution of sentiment and topic frequency demonstrates a tendency in the most frequently mentioned topics to be the most polarized, appearing as a u-shaped curve (Figure 2B). The topic sentiment scores over time appeared to vary around their overall corpus sentiment score and did not significantly change throughout the study period. Consultancy appeared to vary most significantly across the study period. Mental health demonstrated a downward trend toward the latter part of the study period. Good experience, good staff, and operations and surgery had consistently higher sentiment scores across the study period compared with the other topics (Figure 2C).

**Table 2:** Sentiment scale examples.

Sentiment score	Review
−1.0	“Awful treatment in the SAU^a^ area. Avoid using if you can!”
−0.5	“I think there a load of rubbish whenever you ring them they dont answer tried 8 times today what a joke.”
0.0	“Have been attending clinic regularly. Always have eye problems after drops. Despite several attempts to bring this to staff attention it has been dealt with in a dismissive manner. GP^b^ attendance has been necessary. Phone always engaged or left ringing.”
0.1	“All midwives very helpful. An improvement could be that proper beds are provided for partners.”
0.2	“I felt very respected and would like to say thank you.”
0.5	“Excellent result from the procedure performed from the team, my sight is back to what I want. As always (department name) do the business.”
1.0	“The care of all the staff was excellent; and some of them deserved an MBE^c^.”

^a^SAU: surgical assessment unit.

^b^GP: general practitioner.

^c^MBE: Member of the Order of the British Empire.

**Table 3:** Latent Dirichlet allocation–generated topics with their sentiment score (refer to Multimedia Appendix 1 for keywords generated for each cluster).

ID	Topic	Classified reviews, n (%)	Sentiment score
1	Good staff	1579 (7.1)	0.305795
2	Waiting times	1136 (5.1)	−0.006903
3	A&E^a^	2238 (10.1)	0.277627
4	Operations and surgery	2205 (9.9)	0.331010
5	Food and cleanliness	647 (2.9)	0.002680
6	Mental health	1463 (6.6)	0.122655
7	Good experience	2369 (10.7)	0.333013
8	Booking appointments	2272 (10.2)	−0.038362
9	Consultancy and tests	640 (2.9)	0.139792
10	Car parking	1577 (7.1)	0.028805
11	Obstetrics	1075 (4.8)	0.272878
12	Rude staff	1128 (5.1)	−0.110659
13	Procedure and dealing with anxieties	2646 (11.9)	0.330488
14	Paramedic and ambulance	1246 (5.6)	0.250670
N/A^b^	Total	22,221 (100)	0.182695

^a^A&E: accident and emergency.

^b^N/A: not applicable.

### Topic Modeling

A total of 14 clusters were identified from the entire corpus, from which themes were derived manually (Table 3). Reviews were then classified according to their dominant topic (ie, the topic to which the review had a >50% probability of belonging as identified by the LDA model). This threshold was selected to reduce the confounding effect of sentiment analysis by co-occurring opposing sentiments that may be encountered in reviews that contained multiple topics. Associations between topic frequency and geographic distribution and their changes over time were then characterized.

**Figure 3:**
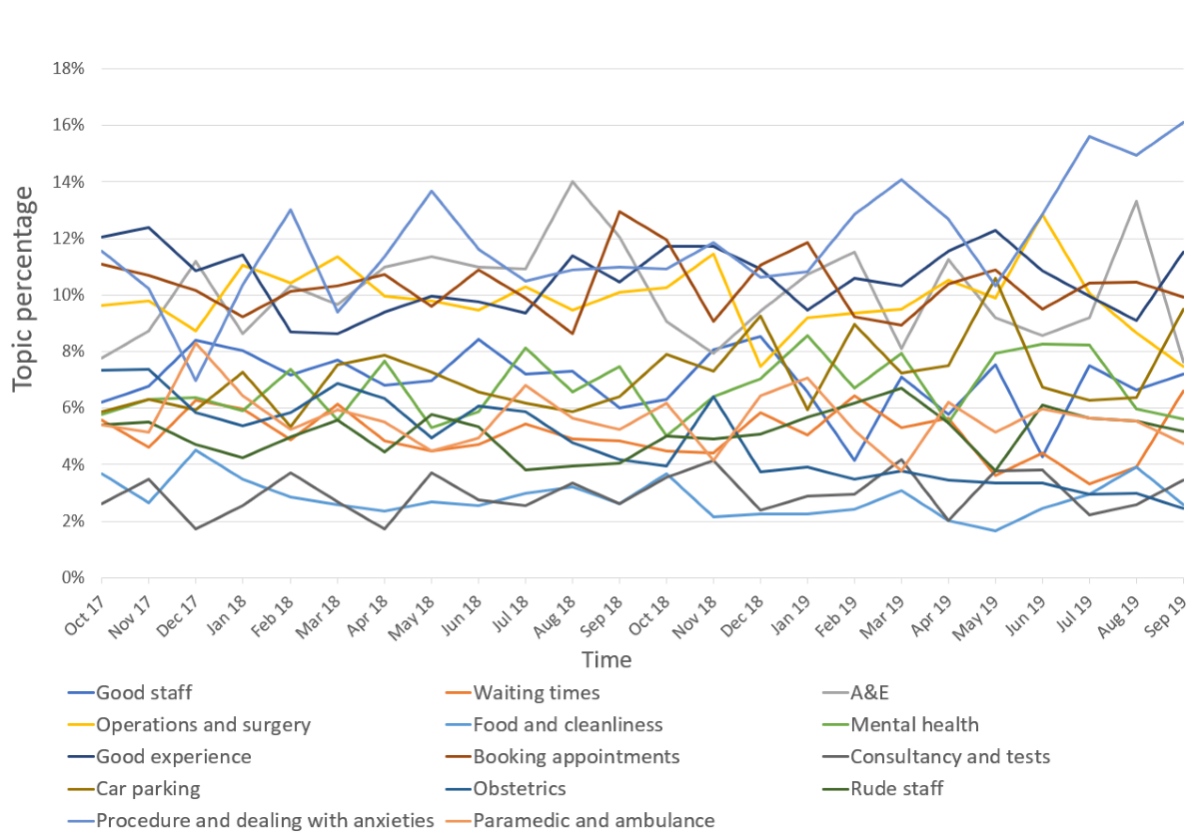
Topics over time. A&E: accident and emergency.

**Figure 4:**
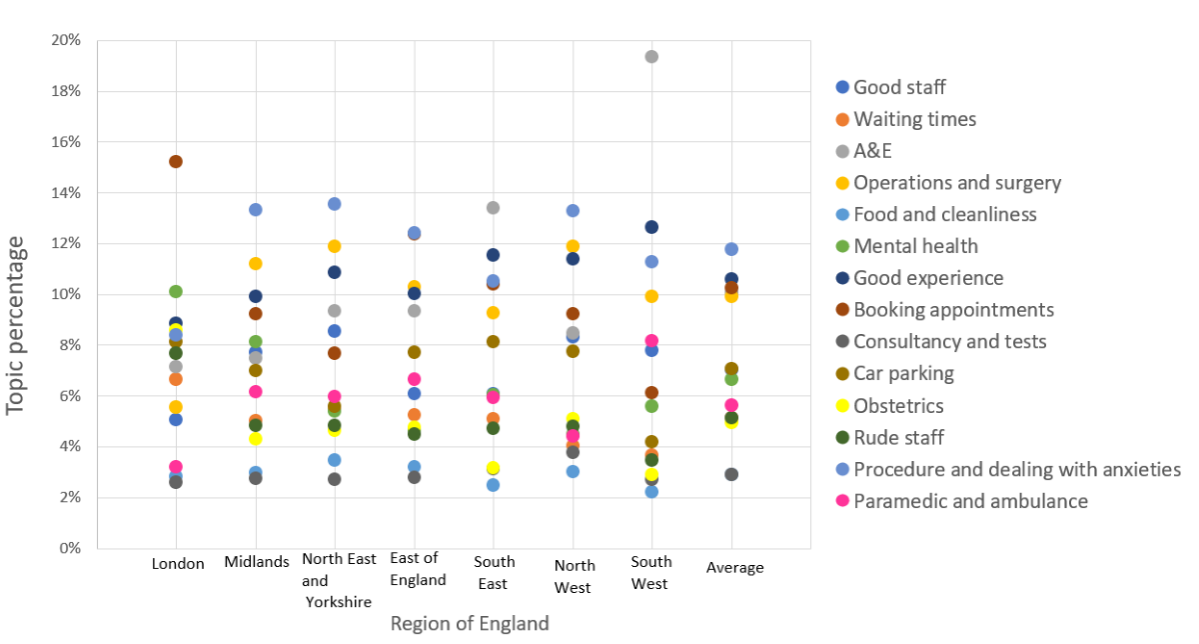
Topic frequency across different regions. A&E: accident and emergency.

In total, 22,221 reviews were classified with a dominant topic, as shown in Table 3, and the topics identified were reviewed and labeled by GFB, who is a medical doctor in the NHS. The most frequent topics identified were *paramedic and ambulance* (topic 14; 1246/22,221, 5.61%), *booking appointment* (topic 8; 2272/22,221, 10.22%), *good experience* (topic 7; 2369/22,221, 10.66%), *operations and surgery* (topic 4; 2205/22,221, 9.92%), and *A&E* (topic 3; 2238/22,221, 10.07%). These topics comprised 46.49% (10,330/22,221) of the labeled reviews. Most topics occurred at a steady rate across the study period. No patterns in that variation in topic frequency were identified; however, *procedure and dealing with anxieties* increased, whereas *obstetrics* decreased in frequency across the study period (Figure 3). Most topics were of similar proportions across all regions in England; however, *waiting time* and *A&E* were proportionately greater in London and the South West, respectively (Figure 4).

### Nested Topic Modeling

Using sentiment scoring, positive-scoring reviews (≥0.2) and negative-scoring reviews (≤0.2) were separated into 2 smaller corpora to undergo topic modeling to obtain new sets of positive and negative topics. The optimal number of topics was decided by creating a model for each number of topics in the range of 5 to 20 and using the coherence score as well as human intuition to determine the best topic model. Positive and negative clusters were identified, and the topics were labeled manually (Tables 4 and 5). The sentiments of both the positive and negative topics demonstrated typical undulations over time, remaining around their average. Regarding positive topics, *admissions* and *surgery* demonstrated rapid increases and decreases back to their average in 2019 and, of the negative topics, *rude*, *booking appointment*, and *food and cleanliness* scored the lowest sentiment.

**Table 4:** Negative-sentiment topics (any review with a sentiment score <−0.2; refer to Multimedia Appendix 2 for keywords generated for each cluster; n=863)

ID	Human-generated name	Reviews, n (%)
1	Mental health	77 (8.9)
2	Care	58 (6.7)
3	Rudeness	65 (7.5)
4	Children	31 (3.6)
5	Pain management	81 (9.4)
6	Waiting for appointment	107 (12.4)
7	Phone	68 (7.9)
8	Cleanliness	49 (5.7)
9	Care	48 (5.6)
10	Booking appointment	159 (18.4)
11	GP^a^	31 (3.6)
12	Results	89 (10.3)

^a^GP: general practitioner.

**Table 5:** Positive-sentiment topics (any review with a sentiment score >+0.2; refer to Multimedia Appendix 3 for keywords generated for each cluster; n=917)

ID	Human-generated name	Reviews, n (%)
1	General care	119 (13)
2	A&E^a^	104 (11.3)
3	Admissions	57 (6.2)
4	Service	93 (10.1)
5	Pediatrics	156 (17)
6	Appointment and consultation	177 (19.3)
7	Dealing with anxieties	74 (8.1)
8	Surgery	137 (14.9)

^a^A&E: accident and emergency.

### Visualization

**Figure 5:**
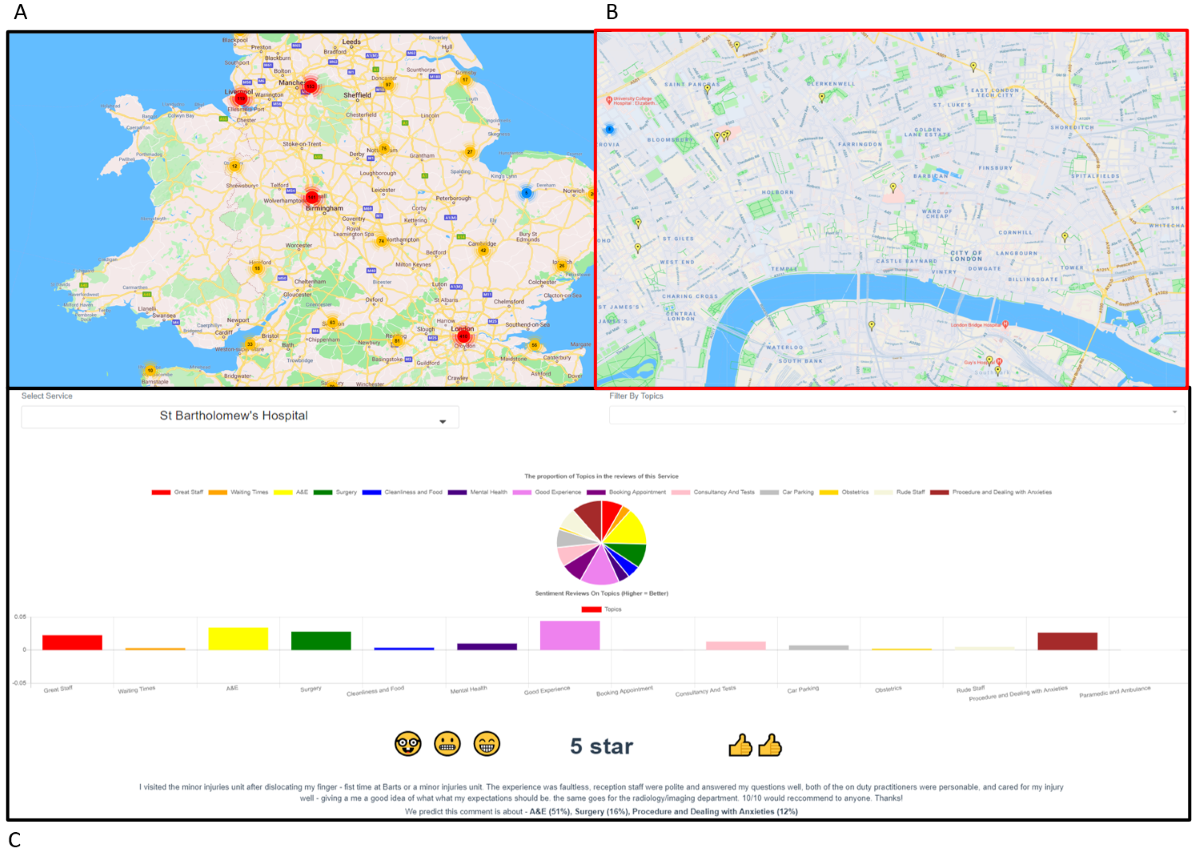
Screenshots of the web-based interface user journey. A: Visualizing NHS services on a map of the United Kingdom; B: A closer view of London and its NHS services; C: The visualization of the sentiment, emotion and topic model analysis. A&E: accident and emergency; NHS: National Health Service.

The NLP analytics were then incorporated into a user-friendly web-based interface that enables the exploration of the data through the graphical user interface (Figure 5). Users can navigate around the country using the Google Maps–based map, which displays color-coded pins that aggregate analytics for areas (Figure 5A). The color of the pins represents the average sentiment score for that service. As the user zooms into an area, the aggregated pins are divided into color-coded pins for individual services (Figure 5B). Clicking on a service reveals a short overview of the service, displaying the average sentiment, the emotions, and the proportion of each topic derived from the reviews of that service (Figure 5B). The emotions were derived from an emotional analysis algorithm applied to each review and accumulated to find the most common emotion. This improves usability as it increases its appeal to the users.

## Discussion

### Principal Findings

The outcomes of the study presented in this paper demonstrate that NLP-based analysis using sentiment analysis and topic modeling can be used to develop an automated solution for analyzing patient feedback and reviewing the performance of a health care center. The results of the sentiment analysis showed that patient feedback can be accurately classified into positive or negative sentiments and the topic modeling approach can be used to identify topics in a patient review. Furthermore, the identified topics were associated with a sentiment based on the results of the sentiment analysis. This study also presented an interface for stakeholders to view and interact with the outcomes of the patient feedback analysis.

In this study, close to 52,000 reviews from 2030 services were considered for analysis over a period of 2 years. The average number of reviews per service was 26 (SD 60.3). This is indicative of a generally low level of patient engagement with providing service feedback. Furthermore, the number of reviews over time shows that patient review activities have seen a consistent downward trend.

A larger corpus of patient reviews can significantly contribute to building analytical models with capabilities to perform more granular analyses such as individual service performance, areas of concern for a selected service, and similar fine-grained analyses. They improve the communication channel between patients and services, and incentives for patient engagement are necessary for increasing the average number of patient reviews and contributing to the development of improved assessment tools.

The identification of a topic based on topic modeling is a useful tool as it helps in understanding the areas of performance and areas of concern for the NHS services. In this study, the most frequent topic was *procedure and dealing with anxieties* (2646/22,221, 11.91%), implying that undertaking a procedure might be an anxious process for patients, and the average sentiment score of 0.33 (SD 0.17; positive score) indicates that the patients are generally happy with the service and care provided by the NHS services.

On the contrary, another frequently reviewed topic was *booking appointments* (2272/22,221, 10.22%; however, the associated average sentiment score of −0.03 (SD 0.18) implies that the patients might be unhappy with the current appointment booking service. Therefore, topic identification, along with the associated sentiment score, allows policy makers to use the learnings from areas of success and potentially apply them to areas of concern to improve patient satisfaction with the NHS services.

The study presented demonstrates a multifactorial analysis that can be performed using topic modeling and sentiment analysis approaches. A large corpus helps in achieving temporal analysis of patient feedback, as demonstrated in Figures 2A-C. Such time-based analysis could shed light on identifying the point when the decline in an area of service occurred and the duration for which the service performed well or poorly. For instance, if the appointment booking service malfunctions frequently, and assuming patients review the appointment service, a temporal analysis could help understand the time and duration for which the service malfunctions and help the service providers diagnose the issue.

Figure 4 illustrates the topic frequency distribution for different regions of NHS centers. It can be observed that topic frequency largely varies from region to region. However, there are some commonalities in the frequently reviewed topics. For instance, the topic of *A&E* was most frequently reviewed across the regions. *Procedure and dealing with anxieties* was another frequently reviewed topic across regions. Region-based, frequently reviewed topics are a piece of beneficial information for policy makers to gain insights into the weak areas of NHS services for each region and, in general, across all regions.

The combined approach of sentiment analysis of topics identified from topic modeling methods helps collect insightful information about health care services. In our study, the sentiment classification of each topic helped in assessing the public’s perception of the NHS services for a given topic, thus reflecting the service quality. With a large corpus of reviews, the sentiment analysis of identified topics over time can potentially explore the links between temporal factors, such as seasons, and patient experience for each topic. The results of this study demonstrate a decrease in sentiment during the winter. A more extensive and diverse data set of patient reviews has the potential to extract links between seasons and specific topics for further causal inferences to be made. For instance, a variation in sentiment scores for a topic across seasons can be analyzed for external causal factors such as influenza outbreaks, pandemics, staff shortages, technological hindrances, political changes, and similar outside factors.

We note that there are limitations to this study; the first one was that we discounted reviews that did not include a dominant topic. This reduced the population size and ignored reviews that might provide valuable insight. Second, both sentiment analysis and topic modeling inevitably have misclassification errors, especially when the user reviews can be misspelled or have a double meaning (sarcasm). The third limitation is that the topics from the topic modeling are influenced by words that carry sentiment; this causes some topics to carry sentimental meaning, such as *good staff*, rather than just the topic itself. Although this did not affect the accuracy of the results, it could cause the topics to be less useful.

Providing patient reviews in an easily inferable format through visualization tools to the public can foster competitiveness to improve among the NHS services. It is essential to provide analytical outcomes in an accessible format to general users to support patient autonomy and decision-making. The visualization tool developed in this study has the potential to rapidly and easily disseminate the findings of this study using maps and graph-based visual analytics. The visualization further enables users to view the feedback of an NHS service based on the key areas identified from the topics.

### Conclusions

This study presents an automated system for the analysis of patient feedback based on NLP techniques and topic modeling. The DSR methodology was adopted for this study to conduct the patient feedback analysis in 3 iterations. In the first iteration, sentiment analysis of the patient feedback corpus was performed followed by a topic modeling–based analysis of the corpus. In the third iteration, the sentiment scores and topics identified were further analyzed to associate the sentiment scores with the topics identified and categorize the corpus into good and bad corpora. Furthermore, we provided a data visualization interface with potential use for policy makers and associated stakeholders to review the performance of health care centers individually as well as by regions and identify the possible causes behind the performance of a health care center. The future work of our study aims to collect and analyze larger patient feedback databases to build more accurate analytical models. We are exploring artificial intelligence–based NLP models to include them in our analysis.

## Appendix

Multimedia Appendix 1 Latent Dirichlet allocation–generated topics with their sentiment score.

Multimedia Appendix 2

Multimedia Appendix 3 Positive-sentiment topics (any review with a sentiment score >+0.2).

## Abbreviations

API: application programming interface
CTM: correlated topic modeling
DSR: design science research
LDA: latent Dirichlet allocation
LSI: latent semantic indexing
NB: naïve Bayes
NHS: National Health Service
NLP: natural language processing
