# Supplementary material for: Automating Large-scale Health Care Service Feedback Analysis: Sentiment Analysis and Topic Modeling Study
Source: JMIR Med Inform. 2022 Apr 11;10(4):e29385. doi: 10.2196/29385 (PMC9039814; doi:10.2196/29385)
Supplement: Multimedia Appendix 1 [file medinform_v10i4e29385_app1.docx]

Latent Dirichlet allocation–generated topics with their sentiment score

| ID | Topic | Key Words | Classified reviews (n) | Sentiment Score |
| --- | --- | --- | --- | --- |
| 1 | Good Staff | 0.010*"son" + 0.010*"care" + 0.009*"staff" + 0.008*"make" + 0.008*"nurse" + 0.008*"daughter" | 1579 | 0.305795 |
| 2 | Waiting Times | 0.010*"hour" + 0.010*"wait" + 0.009*"pain" + 0.007*"doctor" + 0.006*"give" + 0.005*"check" | 1136 | -0.006903 |
| 3 | A&E | 0.012*"ray" + 0.008*"minute" + 0.007*"injury" + 0.007*"visit" + 0.007*"hour" + 0.007*"minor_injury" | 2238 | 0.277627 |
| 4 | Operations and Surgery | 0.015*"operation" + 0.012*"surgery" + 0.011*"ward" + 0.010*"surgeon" + 0.008*"care" + 0.008*"admit" | 2205 | 0.331010 |
| 5 | Food and Cleanliness | 0.013*"ward" + 0.013*"patient" + 0.009*"bed" + 0.007*"nurse" + 0.007*"leave" + 0.006*"staff" | 647 | 0.002680 |
| 6 | Mental Health | 0.011*"mental_health" + 0.009*"service" + 0.006*"support" + 0.006*"year" + 0.005*"issue" | 1463 | 0.122655 |
| 7 | Good Experience | 0.013*"nh" + 0.012*"staff" + 0.009*"care" + 0.008*"treat" + 0.007*"patient" + 0.006*"time" | 2369 | 0.333013 |
| 8 | Booking Appointments | 0.015*"appointment" + 0.014*"call" + 0.010*"phone" + 0.009*"ring" + 0.008*"contact" | 2272 | -0.038362 |
| 9 | Consultancy And Tests | 0.010*"refer" + 0.009*"consultant" + 0.007*"treatment" + 0.007*"result" + 0.007*"test" | 640 | 0.139792 |
| 10 | Car Parking | 0.010*"wait" + 0.009*"patient" + 0.008*"park" + 0.008*"hospital" + 0.008*"time" + 0.007*"hour" | 1577 | 0.028805 |
| 11 | Obstetrics | '0.026*"baby" + 0.025*"midwife" + 0.009*"birth" + 0.007*"labour" + 0.007*"care" + 0.006*"partner" + 0.006*"daughter" + 0.006*"pregnancy" + 0.006*"experience" + 0.005*"labour_ward"' | 1075 | 0.272878 |
| 12 | Rude Staff | 0.009*"speak" + 0.008*"patient" + 0.006*"leave" + 0.005*"nurse" + 0.005*"rude" +0.005*"doctor" | 1128 | -0.110659 |
