# Supplementary material for: Automating Large-scale Health Care Service Feedback Analysis: Sentiment Analysis and Topic Modeling Study
Source: JMIR Med Inform. 2022 Apr 11;10(4):e29385. doi: 10.2196/29385 (PMC9039814; doi:10.2196/29385)
Supplement: Multimedia Appendix 2 [file medinform_v10i4e29385_app2.docx]

Negative-sentiment topics (any review with a sentiment score <−0.2)

| ID | Human Generated Name | Key Words | Number of Reviews |
| --- | --- | --- | --- |
| 1 | Mental Health | 0.025*"service" + 0.019*"mental_health" + 0.016*"make" + 0.015*"care" + 0.013*"feel" + 0.012*"leave" + 0.010*"time" + 0.009*"life" + 0.009*"speak" + 0.008*"suffer" | 77 |
| 2 | Care | '0.038*"staff" + 0.023*"patient" + 0.022*"hospital" + 0.012*"care" + 0.011*"service" + 0.011*"respect" + 0.011*"lack" + 0.011*"work" + 0.010*"experience" + 0.009*"ward"' | 58 |
| 3 | Rudeness | '0.025*"rude" + 0.024*"staff" + 0.017*"doctor" + 0.016*"feel" + 0.016*"make" + 0.015*"nurse" + 0.014*"give" + 0.013*"care" + 0.011*"patient" + 0.011*"hospital"' | 65 |
| 4 | Children | '0.023*"son" + 0.020*"hour" + 0.018*"centre" + 0.018*"time" + 0.017*"pm" + 0.015*"child" + 0.015*"hospital" + 0.014*"walk" + 0.013*"wait" + 0.013*"park"' | 31 |
| 5 | Pain Management | '0.036*"pain" + 0.020*"hour" + 0.020*"wait" + 0.016*"doctor" + 0.015*"hospital" + 0.012*"daughter" + 0.011*"leave" + 0.009*"nurse" + 0.008*"day" + 0.008*"check"' | 81 |
| 6 | Waiting for Appointment | '0.050*"wait" + 0.030*"hour" + 0.020*"appointment" + 0.019*"patient" + 0.019*"time" + 0.016*"minute" + 0.014*"arrive" + 0.013*"doctor" + 0.012*"people" + 0.011*"clinic"' | 107 |
| 7 | Phone | '0.024*"rude" + 0.020*"speak" + 0.017*"call" + 0.016*"patient" + 0.015*"receptionist" + 0.015*"staff" + 0.011*"make" + 0.010*"talk" + 0.010*"person" + 0.010*"phone"' | 68 |
| 8 | Cleanliness | '0.017*"staff" + 0.017*"nurse" + 0.015*"dirty" + 0.015*"ward" + 0.014*"bed" + 0.014*"hour" + 0.014*"toilet" + 0.012*"floor" + 0.012*"room" + 0.011*"put"' | 49 |
| 9 | Care | '0.037*"ward" + 0.032*"hospital" + 0.024*"care" + 0.017*"mum" + 0.015*"day" + 0.014*"mother" + 0.013*"nurse" + 0.012*"treatment" + 0.012*"find" + 0.012*"discharge"' | 48 |
| 10 | Booking Appointment | '0.046*"appointment" + 0.034*"phone" + 0.030*"call" + 0.027*"time" + 0.020*"ring" + 0.019*"answer" + 0.015*"contact" + 0.013*"service" + 0.013*"department" + 0.011*"minute"' | 159 |
| 11 | GP | '0.021*"gp" + 0.017*"appointment" + 0.016*"consultant" + 0.014*"doctor" + 0.013*"letter" + 0.013*"time" + 0.013*"give" + 0.012*"condition" + 0.012*"department" + 0.010*"make"' | 31 |
| 12 | Results | '0.031*"appointment" + 0.028*"week" + 0.024*"month" + 0.017*"result" + 0.017*"wait" + 0.016*"refer" + 0.016*"consultant" + 0.014*"day" + 0.012*"scan" + 0.011*"call"' | 89 |
