# Supplementary material for: Automating Large-scale Health Care Service Feedback Analysis: Sentiment Analysis and Topic Modeling Study
Source: JMIR Med Inform. 2022 Apr 11;10(4):e29385. doi: 10.2196/29385 (PMC9039814; doi:10.2196/29385)
Supplement: Multimedia Appendix 3 [file medinform_v10i4e29385_app3.docx]

Positive-sentiment topics (any review with a sentiment score > +0.2)

| ID | Human Generated Name | Key Words | Number of Reviews |
| --- | --- | --- | --- |
| 1 | General Care | 0.039*"care" + 0.028*"staff" + 0.025*"treat" + 0.024*"hospital" + 0.020*"respect" + 0.020*"nurse" + 0.017*"team" + 0.017*"ward" + 0.017*"would_like" + 0.015*"doctor" | 119 |
| 2 | A & E | 0.021*"hour" + 0.018*"minute" + 0.015*"attend" + 0.014*"visit" + 0.014*"nurse" + 0.012*"ray" + 0.012*"follow" + 0.012*"service" + 0.011*"excellent_service" + 0.010*"wait" | 104 |
| 3 | Admissions | 0.017*"time" + 0.016*"good" + 0.016*"hospital" + 0.015*"wait" + 0.014*"staff" + 0.013*"find" + 0.012*"wonderful" + 0.011*"doctor" + 0.011*"excellent" + 0.011*"appointment" | 57 |
| 4 | Service | 0.019*"give" + 0.017*"staff" + 0.016*"time" + 0.015*"excellent" + 0.014*"service" + 0.014*"hospital" + 0.013*"care" + 0.011*"treatment" + 0.011*"work" + 0.010*"wonderful" | 93 |
| 5 | Paediatrics | 0.041*"staff" + 0.023*"make" + 0.022*"doctor" + 0.021*"care" + 0.018*"amaze" + 0.017*"brilliant" + 0.017*"daughter" + 0.014*"nurse" + 0.012*"midwife" + 0.012*"great" | 156 |
| 6 | Appointment and Consultation | 0.024*"happy" + 0.021*"staff" + 0.020*"appointment" + 0.018*"treatment" + 0.018*"consultant" + 0.018*"visit" + 0.017*"excellent" + 0.013*"department" + 0.013*"impress" + 0.013*"service" | 177 |
| 7 | Dealing with Anxieties | 0.033*"ease" + 0.031*"staff" + 0.029*"put" + 0.028*"nurse" + 0.024*"make" + 0.023*"procedure" + 0.017*"attend" + 0.015*"feel" + 0.014*"visit" + 0.013*"great" | 74 |
| 8 | Surgery | 0.039*"ward" + 0.038*"care" + 0.026*"surgeon" + 0.021*"operation" + 0.019*"nurse" + 0.018*"surgery" + 0.017*"staff" + 0.016*"excellent" + 0.015*"team" + 0.015*"hospital" | 137 |
